# Exploring risk profiles and emergency frequency of purchasers and non-purchasers of personal emergency alarms: a prospective cohort study

**DOI:** 10.1186/s12877-015-0139-4

**Published:** 2015-10-27

**Authors:** Kristen De San Miguel, Gill Lewin, Elissa Burton, Christine Toye, Duncan Boldy, Peter Howat

**Affiliations:** Research Department, Silver Chain Group, Perth, Australia; School of Nursing and Midwifery, Curtin University, Perth, Australia; School of Physiotherapy and Exercise Science, Curtin University, Perth, Australia; School of Nursing, Midwifery and Paramedicine, Curtin University, Perth, Australia; Centre for Nursing Research at Sir Charles Gairdner Hospital, Perth, Australia; School of Public Health, Curtin University, Perth, Australia

**Keywords:** Assistive technology, Personal alarms, Older people

## Abstract

**Background:**

Personal alarms support independent living and have the potential to reduce serious consequences after a fall or during a medical emergency. While some Australian states have government funded personal alarm programs, others do not; but user-pays services are available. Although several studies have examined the profiles of alarm users, little is known about the risk profile of non-users. Specifically, whether there are “at risk” individuals who are unable, or choose not to purchase a service, who experience a home-based emergency in which an alarm could have mitigated an adverse outcome. This study aimed to describe the ‘risk profile’ of purchasers and non-purchasers of alarms; explore the reasons behind the decision to purchase or not to purchase and identify how often emergency assistance was needed and why.

**Methods:**

Purchasers and non-purchasers were followed for one year in this prospective cohort study. Demographic, decision-making and risk factor data were collected at an initial face-to-face interview, while information about emergencies was collected by monthly calls.

**Results:**

One hundred and fifty-seven purchasers and sixty-five non-purchasers completed the study. The risk profiles between the groups were similar in terms of gender, living arrangements, fall history and medical conditions. Purchasers (Mean = 82.6 years) were significantly older than non-purchasers (Mean = 79.3 years), (t(220) = −3.38, *p* = 0.000) and more functionally dependent on the IADL (z = −2.57, *p* = 0.010) and ADL (z = −2.45 *p* = 0.014) function scores. Non-purchasers (Mean = 8.04, SD = 3.57) were more socially isolated with significantly fewer family networks than purchasers (Mean = 9.46, SD = 3.25) (t(220) = −2.86, *p* = 0.005). Both groups experienced similarly high numbers of emergencies, 38.2 % of purchasers and 41.5 % of non-purchasers had at least one emergency where an alarm could have assisted. Main reasons for non-purchase were: cost (77 %), limited alarm range (51 %), no need (39 %) and lack of suitable contacts (30 %).

**Conclusion:**

There are older individuals who are at high risk of an emergency who are choosing, often for financial and lack of family support reasons, not to purchase a personal alarm service. Greater availability of government funded subsidy schemes would enable these individuals to access a service. Increasing the range over which alarms work could increase their appeal to a broader range of older persons living in the community. Future research should consider how strategies that improve social isolation from family and challenge clients’ beliefs about their own health and independence can support increased access to personal alarm services.

## Background

With Australia’s population ageing and the number of people living alone increasing, utilising assistive technology to enable older Australians to age well and productively at home is an area of growing importance. Personal alarms are one form of assistive technology designed to support independent living by enabling people to gain fast assistance in an emergency. Typically, the person accesses the emergency service by pressing the button on the necklace pendant. The pendant acts as a radio transmitter that communicates with a unit in the person’s home which is connected to a 24-h monitoring call centre.

The types of emergencies that personal alarms have significant potential to address are in the main: medical emergencies, such as cardiac or respiratory problems, that require rapid access to assistance, and falls in older people when the individual has difficulty getting up by themselves. A study by Fleming showed that two thirds of people who fell were unable to get up unassisted and that 15 % of all reported falls resulted in the person being on the floor for an hour or more [1]. A “long lie” after a fall has been shown to be associated with poor outcomes including increased risk of hospital admission, poor functional recovery, subsequent moves into long term care and even death [1, 2]. In Gurley’s 1996 study, only 38 % of people who were admitted to hospital after a fall were able to return to independent living [2]. Additionally, suffering from a fall can affect a person’s confidence, causing them to restrict their daily activities out of fear of falling again [3]. This can lead to functional decline and ultimately impact on their ability to remain living independently [3]. Research on the impact of personal alarms has shown that apart from providing people with faster assistance in emergencies they can also provide a sense of security and reduce anxiety about falling [4], reduce anxiety for the person’s family [5], increase confidence in performing everyday activities and extend the time people are able to remain living independently in their own home [4].

Because of the potential of personal alarms to reduce serious consequences after a fall or during a medical emergency, some States in Australia, as well as the Department of Veterans’ Affairs, have funded personal alarm programs for individuals assessed as being at high risk of having an emergency. User-pays services are however available in all States. While a number of studies have described the profiles of alarm users [4, 6, 7], and found the rate of uptake in different communities of older persons to be low [8], those who do not use/purchase alarms are an under researched group. Some studies have identified reasons for non-use, such as: cost [5, 9], lack of perceived need [5] lack of awareness in the community [9] and being unattractive in appearance [10]. However, little is actually known about individuals who choose not to purchase an alarm; especially whether they would be assessed as less at risk of having an emergency than purchasers and how often they experience emergency situations in which an alarm would have been likely to reduce any negative impact.

The objectives of this study were to: describe the ‘risk profile’ of purchasers and non-purchasers of alarms; explore the reasons behind the decision to purchase and not to purchase an alarm and identify how often emergency assistance was needed and why.

## Methods

### Study design and participants

This study was a prospective cohort study conducted in Perth, Western Australia between February 2011 and June 2013. To be eligible individuals or a family member on their behalf, needed: to have purchased, or enquired about purchasing a personal alarm; be aged 65 years or older; be English speaking; and have no diagnosis of dementia.

The target sample size was 200 in each of the two groups (purchasers and non-purchasers) allowing for an attrition rate of 20 %. Calculation of the sample size was based on historical data of the number of calls for assistance made per month from the total population of clients from the personal alarm service. The sample size was constructed to detect an effect size of 5–10 %, with 80 % power and a 5 % level of significance.

### Recruitment

Participants were recruited via the personal alarm service of a large community health and aged care organisation in Western Australia. Individuals making enquiries about purchasing an alarm, who met the eligibility criteria, were asked if their contact details could be provided to the researchers together with the date of the enquiry. When an individual subsequently purchased an alarm, or after six weeks if they had not purchased an alarm, they were sent a letter and information statement explaining the study. The letter was followed up with a phone call asking whether they were willing to participate. If they agreed, arrangements were made for the researcher to visit them in their home where written consent was obtained before conducting the initial interview.

Recruitment of non-purchasers was particularly slow as the majority of enquiries translated into purchases or enquiries were being made on behalf of someone else and it was not possible to ask them to identify the person concerned without their permission. Due to time and funding constraints it was therefore necessary to curtail recruitment before the target sample size was achieved.

### Data collection

All participants took part in an initial face-to-face interview where information on demographics, the decision-making process, reasons for purchase and non-purchase and risk factors were collected. Potential risk factors were identified from the literature and from items included in eligibility assessments for government subsidised Personal Alarm schemes, which included living arrangements, fall history, prescription medications and medical conditions. In addition, standardised tools were used to collect data on falls efficacy (Modified Falls Efficacy Scale, MFES) [11]; social isolation (Lubben Social Network Scale, LSNS) [12]; personal wellbeing (Personal Wellbeing Index, PWI) [13] and functional dependency (Modified Barthel Index, Activities of Daily Living, ADL) [14] and the Lawton and Brody Scale, Instrumental Activities of Daily Living, IADL [15] with the scoring modified to increase according to the amount of assistance required on a task [16]. At the end of the interview, participants were given a diary and asked to record any emergencies as soon as possible after they happened, to assist with recall at the monthly follow-ups.

Participants were then contacted by telephone each month for 12 months to collect information on the number and nature of emergencies that had occurred. Emergencies were defined as any situation or event where an individual required immediate medical assistance; required assistance from another person to get up after a fall; or, spent more than 10 min on the floor unable to get up. As the research was examining circumstances in which a personal alarm may have been used, only information about situations occurring in the participants’ own homes was collected.

After 12 months involvement in the study, participants were mailed a survey and asked to complete and return it in the stamped addressed envelope provided. This sought to gain a more detailed understanding of the factors identified in the initial interview as being influential in the decision to purchase or not to purchase an alarm. It also aimed to explore any changes in falls efficacy, social isolation and personal wellbeing, experienced during the study period. These latter data will be considered in a subsequent journal article.

### Data management and analysis

All data were checked for completeness on an ongoing basis and entered into a study database at study end. Analyses were performed using STATA version 12 [17]. Data were initially examined for normality of distribution. *T*-tests, chi-square tests or other appropriate non-parametric tests, were performed depending on the type and distribution of the variable being examined, to determine any differences between the groups. Responses to the open-ended questions in the initial interviews were transcribed verbatim. The key emerging themes behind the decision to purchase or not to purchase an alarm were identified, coded and then summarised. These themes were then used to develop the response categories used in the 12-month survey which required participants to rate how important each of these reasons were.

### Alarm functioning and costs

The personal alarms described in this study are small water resistant pendants that are worn around the neck. The pendant acts as a radio transmitter that communicates with a unit in the person’s home which is connected to a 24-h, 7 days a week monitoring centre center. All calls are answered by a trained operator who will view the person’s medical history, determine the appropriate response and stay on the line until help arrives. On commencing the service, individuals nominate several contacts (family or friends) that the monitoring centre can call on during an emergency situation.

In Australia alarms may be purchased for approximately $600 (equipment and installation fee) with a monthly monitoring fee of $19. They may also be rented for an initial cost of $216 with a monthly monitoring fee of $36.

### Ethics

Approval to conduct the project was given by the Human Research Ethics Committees of the University and the alarm provider organisation.

## Results

**Fig. 1:**
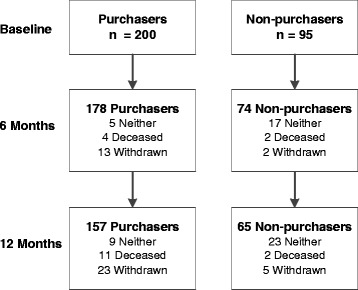
Participant flow through the study

Two hundred purchasers and 95 non-purchasers participated in the study. Results are presented for participants with 12 months of complete data (157 purchasers and 65 non-purchasers). Figure 1 shows the participant flow through the study, as some clients withdrew, died or others changed groups (by either purchasing an alarm at some point during the study or ceasing their alarm service) and therefore were not counted in either group (neither).

At twelve months there were 9 purchasers and 23 non-purchasers considered to be in the neither group. Of the nine purchasers, six people discontinued the service because they moved to residential care, two felt they didn’t need the alarm anymore and one because it was too expensive. Of the 23 non-purchasers that decided to purchase an alarm at some point during the study, four changed their mind because of family pressure, four after finding a cheaper alarm service, four were unable to afford it initially and had to save up, four needed more time to find appropriate people to list as contacts or do further research, three had a fall, two were unsure about their future living arrangement, one had a partner who passed away and one was prompted after a change in their health status following surgery.

### Participant profiles

Table 1 outlines the demographics and participant risk profiles. Purchasers and non-purchasers were similar in terms of gender, education and living arrangements with over 70 % of participants in both groups being female, living alone and having completed at least a secondary education. There was a significant difference in age with purchasers (Mean = 82.6 years) being on average 3.3 years older than non-purchasers (Mean = 79.3 years), (t(220) = −3.38, *p* = 0.000).

**Table 1:** Demographics and risk characteristics

Demographics	Purchasers n (%/SD)	Non-purchasers n (%/SD)	*P*-value
Age (Yrs)	82.63 (SD 6.7)	79.35 (SD 6.3)	*0.000*
Gender (% Female)	129 (82.2 %)	47 (72.3 %)	*0.099*
Living arrangement (% Lives Alone)	119 (75.8 %)	53 (81.5 %)	*0.351*
Receiving formal home care services	90 (57.3 %)	40 (61.5 %)	*0.562*
Level of education			
Primary	36 (22.9 %)	10 (15.4 %)	*0.053*
Secondary	81 (51.6 %)	28 (43.1 %)	
Tertiary	40 (25.5 %)	27 (41.5 %)	
Medical conditions			
Arthritis	105 (66.9 %)	39 (60.0 %)	*0.329*
Heart condition	46 (29.3 %)	15 (23.1 %)	*0.345*
Respiratory condition	46 (29.3 %)	20 (30.8 %)	*0.827*
Diabetes	33 (21.0 %)	15 (23.1 %)	*0.735*
Depression	21 (13.4 %)	16 (24.6 %)	*0.041*
Osteoporosis	54 (34.4 %)	20 (30.8 %)	*0.602*
Prescription medications			
5 or more	85 (54.5 %)	35 (53.8 %)	*0.931*
Functionality			
Instrumental Activities of Daily Living (IADL)^a^	12.54 (12,6)	11.08 (10,3)	*0.010*
Activities of Daily Living (ADL)^b^	11.02 (10,2)	10.58 (10, 1)	*0.014*
Fall history			
Modified Falls Efficacy Scale (MFES)^c^	8.50 (8.8, 2)	8.40 (8.8, 2.1)	*0.266*
Fallen in past 12 months	97 (61.8 %)	40 (61.5 %)	*0.973*
Past fall resulted in injury requiring medical attention	72 (74.2 %)	26 (65.0 %)	0.277
Past fall resulted in lie over 30 min	31 (32.0 %)	10 (25.0 %)	*0.445*
Worried about falling	70 (44.6 %)	31 (47.7 %)	*0.672*
Restricts activity because worried about falling	52 (33.1 %)	17 (26.1 %)	*0.307*
Social contact			
Lubben Social Network Scale (LSNS)^d^	16.9 (5.82)	16.01 (5.87)	*0.292*
Lubben Social Network Scale : Family subset	9.46 (3.25)	8.05 (3.6)	*0.005*
Lubben Social Network Scale: Friend subset	7.46 (4.0)	7.97 (3.7)	*0.390*
Personal wellbeing			
Personal Wellbeing Index (PWI)^e^	80.2 (82.8, 18.6)	75.46 (78.6, 18.6)	*0.032*

^a^IADL: Mean total score ranges from 0 to 30 with higher scores indicating higher dependency

^b^ADL: Mean total score ranges from 1 to 32 with higher scores indicating higher dependency

^c^MFES: Mean total score ranges from 0 to 10 with higher scores reflecting more confidence, less fear of falling

^d^LSNS: Mean total score ranges from 0 to 30 with higher scores indicating more family and friendship ties. Family and Friend Subset scores range from 0 to 12

^e^PWI: Mean total score ranges from 0 to 100 with higher scores representing higher subjective wellbeing

Approximately 60 % of participants in both groups were receiving at least one formal home care service. There were no differences in the type of services received, with the majority in both groups receiving relatively low care services. Over 70 % of both groups were receiving domestic assistance services only and less than 17 % were receiving services for personal care tasks.

Most individuals in both groups had more than one chronic health problem and were taking an average of five prescription medications per day. Around 60 % of participants in both groups had arthritis, about a third had osteoporosis and or a respiratory condition and over 20 % in both groups had a heart condition and or diabetes. The only significant difference in medical conditions between the groups was that more non-purchasers reported having depression (χ^2^(1, N = 222) =4.18, *p* = 0.041). The IADL (z = −2.57, *p* = 0.010) and ADL (z = −2.45 *p* = 0.014) function scores showed a significant difference between the groups with the non-purchasers having lower average scores on each scale, indicating that they were more independent. Specifically, non-purchasers were more independent in the tasks of shopping, travelling around outside their home and climbing stairs.

There were no differences between the groups in terms of fall history or in their confidence in performing every day activities without worrying about falling, as measured by the MFES. Over 60 % of participants in both groups had had a fall in the previous 12 months and nearly a third had been on the floor unable to get up for over 30 min. Just under half were worried about falling and roughly a third restricted their daily activities because they were worried about falling.

There was a significant difference in the family subset scale of the Lubben Social Network Scale with non-purchasers (Mean = 8.04, SD = 3.57) being more socially isolated with significantly fewer family networks than purchasers (Mean = 9.46, SD = 3.25) (t(220) = −2.86, *p* = 0.005). Non-purchasers were also less likely to have anyone visit them more than once a week (χ^2^(2, N = 171) =13.47, *p* = 0.001). Non-Purchasers (Mean =75.46, SD 15.47) also scored significantly lower on the personal wellbeing index than purchasers (Mean =80.17, SD 13.37) (t(216) = −2.25, *p* = 0.025).

### Decision to purchase

For purchasers the reasons rated as most important for obtaining the alarm were fear of falling and not being able to get up (89 %), living alone (83 %) and because family wanted them to have one (80 %). For non-purchasers (77 %), cost was the major reason for deciding not to purchase. Other main reasons included the alarm system not having a large enough range (51 %), not thinking they needed it (39 %) and not having any family or friends to list as emergency contacts (30 %) (Table 2). In terms of family involvement, purchasers (*n* = 140, 70 %) were significantly more likely than non-purchasers (*n* = 37, 39 %) to have had family involvement when considering whether to purchase an alarm or not. The level of family involvement varied from finding out about the alarm and passing on information, to organising the purchase and having the alarm installed.

**Table 2:** Reasons for purchase and non-purchase

	Not important	Neither	Very important	Total
Reasons for purchase				
Fear of falling and not being able to get up	7 (5.5 %)	7 (5.5 %)	113 (89.0 %)	127 (100 %)
You live alone	15 (12.1 %)	6 (4.8 %)	103 (83.1 %)	124 (100 %)
Family wanted you to get one	11 (8.6 %)	14 (10.9 %)	103 (80.5 %)	128 (100 %)
Medical condition/health reason	18 (15.6 %)	14 (12.2 %)	83 (72.2 %)	115 (100 %)
Security/fear of intruders	35 (35.6 %)	21 (20.8 %)	45 (45.5 %)	101 (100 %)
Family living interstate or a long way away	44 (49.4 %)	14 (15.7 %)	31 (34.8 %)	89 (100 %)
Reasons for non-purchase				
Cost	2 (4.5 %)	8 (18.2 %)	34 (77.3 %)	44 (100 %)
Did not think you needed it	5 (15.1 %)	15 (45.5 %)	13 (39.4 %)	33 (100 %)
Thought the alarm was unattractive	23 (71.9 %)	7 (21.9 %)	2 (6.2 %)	32 (100 %)
Had no-one to list as an emergency contacto	15 (45.5 %)	8 (24.2 %)	10 (30.3 %)	33 (100 %)
Felt it would take away my independence	25 (78.1 %)	3 (9.4 %)	4 (12.5 %)	32 (100 %)
Was unsure how the alarm worked	14 (45.2 %)	11 (35.5 %)	6 (19.3 %)	31 (100 %)
Thought alarm would be uncomfortable to wear	17 (53.1 %)	12 (37.5 %)	3 (9.4 %)	32 (100 %)
Alarm did not have a big enough range	12 (38.7 %)	3 (9.7 %)	16 (51.6 %)	31 (100 %)

### Emergencies

Over the 12 months of the study there were 154 emergencies with 38 % of purchasers and 41 % of non-purchasers experiencing at least one. For 57 % of purchasers and 63 % of non-purchasers these emergencies required hospitalisation.

Falls were the most common type of emergency for both groups contributing to 49 % of all emergencies. Other emergencies, in descending frequency, included respiratory difficulties (11 %), experiencing extreme pain (related to stomach, back or kidney) (8 %), feeling unwell (7 %), feeling faint (6 %) and heart problems (4 %). The full range of emergencies by group are shown in Table 3. There were no significant differences between the two groups in the numbers and types of emergencies experienced.

**Table 3:** Emergency type

Emergency type	Purchasers	Non-purchasers	Total
Fall	39 (47.0 %)	37 (52.1 %)	76 (49.3 %)
Respiratory (difficulty breathing, coughing fit, asthma attack)	7 (9.6 %)	10 (14.1 %)	17 (11.0 %)
Experiencing extreme pain (stomach, back, kidney)	9 (10.8 %)	4 (5.6 %)	13 (8.4 %)
Feeling generally very ill	7 (8.4 %)	4 (5.6 %)	11 (7.1 %)
Passed out/fainted/dizzy	2 (2.4 %)	7 (9.8 %)	9 (5.8 %)
Heart problems (chest pains, heart attack, high BP)	4 (4.8 %)	2 (2.8 %)	6 (3.9 %)
Sudden loss of function (leg collapsed, pinched nerve)	3 (3.6 %)	3 (3.7 %)	6 (3.9 %)
Severe swelling (cellulitis, swollen leg)	1 (1.2 %)	3 (3.7 %)	4 (2.6 %)
Suffered deep cut or open wound	3 (3.6 %)	1 (1.4 %)	4 (2.6 %)
Vomiting/nausea	4 (4.8 %)	0 (0.0 %)	4 (2.6 %)
Panic attack	1 (1.2 %)	1 (0.0 %)	2 (1.3 %)
Allergic reaction	1 (1.2 %)	2 (0.0 %)	3 (1.9 %)
Choking	1 (1.2 %)	3 (0.0 %)	4 (2.6 %)
Memory loss/confusion	1 (1.2 %)	4 (0.0 %)	5 (3.2 %)
Total	83 (100 %)	71 (100 %)	154 (100 %)

## Discussion

This study found many similarities between the participants who had and had not purchased an alarm service, in particular their risk profiles and the numbers of emergencies experienced during the follow up period. There were however, a few notable differences. Non-purchasers were slightly younger, less functionally dependent, had less family support and had lower personal wellbeing than the purchasers. By far the most common reason for not having purchased an alarm was cost.

Both groups of participants were similar, demographically and in terms of risk profile, to the users of personal alarms described in previous studies. The majority were women, in their 80′s, living alone, had fallen in the previous 12 months, had multiple medical conditions, were receiving low level formal home care services and took an average of five medications per day [7, 6, 18].

Our findings that the non-purchasers were younger and less functionally dependent than purchasers are consistent with Nyman et al’s 2012 findings that age and greater difficulty with activities/instrumental activities of daily living were the only significant predictors at the multivariate level for use of a personal alarm [8]. Similarly to Nyman and colleagues we did not find social isolation to be significantly associated with use of an alarm, but rather our study found that being socially isolated from family appeared to reduce the likelihood of purchasing an alarm. Non-purchasers were less likely to have family support and be visited by anyone in their home more than once a week. They were also much less likely than purchasers to have had family involvement when first considering an alarm. Over 80 % of purchasers reported that one of the most important reasons for deciding to purchase an alarm was because family wanted them to have one. If non-purchasers are more socially isolated from family, they may have experienced less pressure or encouragement to purchase an alarm and consequently they may have also been less likely to have someone available to assist with the cost.

Despite being younger and less functionally dependent, the non-purchasers experienced as high a rate of emergencies as their older and more dependent counterparts who purchased the service. The types of emergencies experienced were also indistinguishable and an alarm would have been as useful to the non-purchasers as it was to the purchasers.

The majority of non-purchasers wanted to own an alarm but felt unable to afford it. Cost has been reported as a barrier to obtaining a personal alarm in previous research [5, 19]. There are subsidised alarms schemes in Australia, but as in other countries, they vary in criteria, wait times and in the proportion of alarm expenses that are covered [20]. Some cover the initial costs of equipment; others cover the ongoing fees associated with monitoring the alarm, while others may only provide a one off partial reimbursement that does not actually cover either the total cost of the equipment or the ongoing monitoring. In Western Australia, where the only subsidised scheme available at the time of this research was that of the Department of Veterans’ Affairs, most alarms were paid for by the older person themselves or their family. Increased availability of an all-inclusive government funded subsidy scheme would have allowed more equitable access to alarms for those people at risk of a home based emergency.

The limited range, or having to be within 50 m of the alarm console for it to work, was also rated as an important factor in influencing the non-purchase decision for many. They talked about wishing to walk to the local shops or park and for the alarm to be effective during these activities. Whilst most traditional alarms do not have this capability, some companies are already developing systems that have GPS tracking and support wireless voice communication with an operating centre and therefore can be taken out of the home [20]. Increasing the functionality to be able to be used outside the home will appeal to a broader range of users and could assist in facilitating and increasing uptake in the wider community.

Just over a third of non-purchasers reported that they did not think they needed the alarm. Lack of perceived need has been reported previously [5, 19] and is a more difficult barrier to address. As identified by Johnston et al. [19], there are people who are at risk of falls but continue to perceive that they are at low or no risk. Further research is needed to identify those strategies that are most effective in challenging people’s beliefs about their own health and independence and in promoting how the use of assistive technology can enhance, rather than undermine, independence [19]. General Practitioners could well play an important role in assisting their patients to recognise their risk profile and their capabilities and in encouraging them to adopt strategies, such as using a personal alarm, that will enable them to optimise their independence.

A third of non-purchasers also reported that one of the important reasons for not purchasing the alarm service was that they did not have any family or friends to list as suitable emergency contacts. Whilst many alarm services usually have alternative options such as using emergency or other services as contacts, people had the perception that they were not able to have an alarm if they had no contacts to list. This is another illustration of how being socially isolated influences the decision to purchase an alarm and highlights the importance of the easy availability of comprehensive information about alarm use and requirements, particularly with regards to contacts.

Another study reported reasons for non-use due to alarm design or appearance [10]. However, this was not an important factor in this study with less than 10 % reporting that the alarm being uncomfortable to wear or looking unattractive were important reasons in their decision not to purchase an alarm.

### Limitations

Whilst participants were asked about their decision to purchase or not to purchase at the initial interview, these data were qualitative in nature. These key reasons identified for purchase and non-purchase were then rated for importance retrospectively in the 12 month follow-up survey which meant the sample size was reduced to only those responding at 12 months and people had to recall what was important in their decision 12 months earlier. Subsequent experiences during that year may have influenced their responses as to what was important.

As shown in the results the number of participants in the non-purchasers group did not reach the target sample size. Therefore it is possible that results that were approaching statistical significance may, with a larger sample, have reached significance. Review of all variables have shown that the only variable that was approaching significance and likely to have been affected by a type two error due to the study being underpowered for this variable was level of education.

## Conclusions

There are older individuals who are at high risk of an emergency who are choosing, often for financial and lack of family support reasons, not to purchase a personal alarm service. Greater availability of government funded subsidy schemes would enable these individuals to access a service. Increasing the range over which alarms work could increase their appeal to a broader range of older persons living in the community. Future research should consider how strategies that improve social isolation from family and challenge clients’ beliefs about their own health and independence can support increased access to personal alarm services.

## Abbreviations

MFES: modified falls efficacy scale
LSNS: lubben social network scale
PWI: personal wellbeing index
ADL: activities of daily living
IADL: instrumental activities of daily living

## References

[1] Fleming J, Brayne C (2008). Inability to get up after falling, subsequent time on floor, and summoning help: prospective cohort study in people over 90. BMJ.

[2] Gurley RJ, Lum N, Sande M, Lo B, Katz MH (1996). Persons Found in their Homes Helpless or Dead. New Engl J Med.

[3] Zijlstra G, Van Haastregt J, Van Eijk J, Rossum E, Stalenhoef P, Kempen G (2007). Prevalnce and correlates of fear of falling, and associated avoidance of activity in the general population of community-living older people. Age Ageing.

[4] De San MK, Lewin G (2008). Personal emergency alarms: What impact do they have on older people’s lives?. Australas J Ageing.

[5] Mann WC, Belchior P, Tomita RM, Kemp BJ (2005). Use of Personal Emergency Response Systems by Older Individuals with Disabilities. Assist Technol.

[6] Levine A, Tideiksaar R (1995). Personal emergency response systems: factors associated with use among older persons. Mt Sinai J Med.

[7] Heinbuchner B, Hautzinger M, Becker C, Pfeiffer K (2010). Satisfaction and use of personal emergency response systems. Z Gerontol Geriatr.

[8] Nyman SR (2012). R VC. Use of personal care alarms among community-dwelling older people. Ageing Soc.

[9] Berstein M (2000). “Low-Tech” Personal Emergency Response Systems Reduce Costs and Improve Outcomes. Manag Care Q.

[10] Davies KN, Mulley GP (1993). The views of elderly people on emergency alarm use. Clin Rehabil.

[11] Hill K, Schwarz J, Kalogeropolous A, Gibson S (2006). Fear of Falling Revisited. Arch Phys Med Rehab.

[12] Lubben J, Blozik E, Gillmann G, Iliffe S, von Renteln KW, Beck J (2006). Performance of an abbreviated version of the Lubben Social Network Scale among three European community-dwelling older adult populations. Gerontologist.

[13] The International Wellbeing Group (2013). Personal Wellbeing Index-Adult.

[14] Colin C, Wade D, Davies S, Horne V (1988). The Barthel ADL Index: A reliability study. Int Disabil Stud.

[15] Lawton M, Brody E (1969). Assessment of older people: Self-maintaining and instrumental activities of daily living. Gerontologist.

[16] Calver J, Lewin G, Holman C (2002). Reliability of a primary, generic assessment instrument for home care. Australas J Ageing.

[17] StataCorp (2011). Stata Statistical Software: Release 12.

[18] Hyer K, Rudlick L (1994). The Effectiveness of Personal Emergency Response Systems in Meeting the Safety Monitoring Needs of Home Care Clients. J Nurs Admin.

[19] Johnston K, Grimmers-Sommers K, Sutherland M (2010). Perspective on use of personal alarms by older fallers. Int J Gen Med.

[20] Hessels V, Le Prell G, Mann WC (2011). Advances in Personal Emergency Response and Detection Systems. Assist Technol.

